# Inoculating the Ground

**Published:** 1905-01

**Authors:** 


### Inoculating the Ground.

We have become so accustomed to regard the omnipresent micro-organism as a source of unmixed evil that his good works are allowed to go unnoticed, or, at the most, to receive but scant attention. His beneficent offices are given some credit in the manufacture of fermented liquors and in the purification of streams, but if the statement had been made a decade ago that micro-organic life would reclaim to agriculture thousands of acres of barren and impoverished land, and add to the wealth of the country untold millions of dollars, it would have been given the credence of a tale from "Arabian Nights," and yet perfectly controlled and practical experiment has demonstrated beyond the shadow of a doubt that the ground can be inoculated with bacteria and enriched to a truly marvelous degree.*

It might perhaps be well, before taking up this extraordinary discovery in detail, to bring to mind a few facts in relation to plant life. In the first place, it is generally accepted that one of the most important, if not the most important, element of plant-food is nitrogen, which is absorbed from the soil mainly through the roots. It is equally well known that successive crops of grain will in time so drain nitrogen from a soil as to render the richest land poor and unproductive. This sterility was to a certain extent overcome by supplying the nitrogen artificially by means of impregnating the earth with manure and fertilizer—a method at once tedious and expensive. Every year thousands of tons of nitrate are imported into this country, notwithstanding which the soil, especially in Eastern sections, is constantly becoming more and more unproductive, so much so, indeed, that many scientists have predicted "a nitrogen famine," or, in other words, a grain famine, at no distant day. Yet all who have given the subject any thought recognized the fact that all about us, in the air we breathe, there exists a veritable sea of nitrogen, which if it could be captured, and, as it were, poured into the ground, would form an inexhaustible supply of fertilizer. This seemed like a simple problem, but with the first step, contributed by farmers centuries ago, in that they noticed that after a crop of peas or any

*Grosvenor in October Century.

of the leguminous plants a heavier yield of grain was noted, and which brought about the establishment of the old rule of rotation of crops, little was accomplished in solving it until a few years ago.

The reason why certain plants enrich the soil, while others only succeed in making the yield more unproductive, was undetermined until a few years ago, when Professor Nobbe, of Tharandt, found that the legumens obtained their nitrogen directly from the atmosphere. It was further noted that these legumens were capable of absorbing considerably more of the nitrogen than could be subserved for their own growth, and that this excess was thriftily deposited in the soil. In other words, what the grain removed the legumens resupplied, explaining readily the reason for the rotation of crops.

Now, if the roots of a healthy bean-plant are examined, there will be found upon them a number of nodules which give them a diseased appearance, as if they had been bitten by insects or worms, and all plants of the so-called leguminous order are supplied with these excrescences, which vary in size fron a pin-head to that of a potato. Careful investigation showed that the larger these tubercles the more the plant flourished, and *vice versa*, from which it was concluded that they had not a little to do with the nutrition of the plant. Microscopic examinations demonstrated that the nodules were literally composed of bacteria, which on further testing were found to be constantly absorbing free nitrogen from the atmosphere and changing it into a form easier for the plant's digestion. These micro-organisms were called the nitrogen-fixing bacteria, and were found to exist not alone in connection with the roots of the plants, but in the soil as well, where they attached themselves to the roots and multiplied enormously. The tubercles, in bacteriological parlance, then, were colonies of bacteria.

It was next found that soil that did not contain the nitrogen-fixing bacteria could not be made to grow a crop of any of the leguminous plants, and, as said before, the larger the tubercles the more flourishing the growth of the plants. "One might thus define a tubercle as a little factory where millions of tireless, infinitesimal workers are separating the nitrogen in the air and converting it into plant-food." It was evident, then, that if these bacteria could be cultivated artificially, in the laboratory, a soil inoculated with them could be made to produce an enormous crop of the legumens and the following year a proportionately larger amount of grains. This Professor Nobbe proceeded to do, but his nitrogen, when put upon the market, proved a failure, though it gave to its discoverer a large fortune. It was found that the bacteria burned themselves out, as it were, without producing a single nodule.

Dr. George T. Moore, an American, in studying the point at issue, came to the conclusion that Nobbe had not cultivated his bacteria in the right way; that he had, so to speak, pampered them too much, and that when left to their own resources outside the laboratory, unable to shift for themselves—i. e., to hunt out and convert nitrogen—they were helpless. Following up this idea, he was able to grow bacteria with from five to ten times the power of the original germs for fixing free nitrogen, and which, when left to themselves, flourish luxuriantly. Leguminous plants inoculated with the new growth developed tubercles of the largest size and grew splendidly in the poorest soil. The problem was solved —not only solved, but to such a degree of perfection that seeds previously soaked in Moore's solution will grow well even when planted in quartz sand that had been previously heated to red heat to rid it of all nitrates. The next step was to be able to send them to various sections of the country without impairing their vitality. This proved to be simple. Moore found that when the solution in which the bacteria were growing was soaked up by ordinary absorbent cotton, and the latter dried, the bacteria could be revived merely by reintroducing the cotton to a liquid medium, so that these dried cotton pledgets could be mailed to any part of the world and arrive in good condition.

Even a short article of this kind should not be written without a tribute to Professor Moore, not alone for his careful work, but in that, having patented his discovery, as he had a perfect right to do, he deeded the patent to the Department of Agriculture, in trust for the American people—a piece of generosity that in this day remains almost unexampled, as he had a large fortune within his grasp. At present the Department of Agriculture will send to applicants *gratis* enough bacteria to inoculate several acres, and the directions for using, accompanying each package, are so simple that a child could understand them. What has been the practical result? Not alone an enormous increase in the crop of legumes, but the latter have left enough nitrogen in the soil to multiply many times over the crop of grain or potatoes planted the following year, as can be readily seen by a glance at the subjoined table:

| | Original Yield per Acre. | Yield per Acre After Inoculated Crop. |
|---|---|---|
| Cotton .... | 932. pounds. | After red clover, 1,304 pounds. |
| Potatoes ... | 67.8 bushels. | After crimson clover, 102.2 bushels. |
| Oats ....... | 8.4 " | After velvet beans, 33.6 bushels. |
| Rye........ | 4.5 " | After peas, 23.5 bushels. |
| Wheat..... | 18.6 " | After melilotus, 26.9 bushels. |

Fortunately, every section of our country and every country will benefit by this wonderful discovery, and the wealth accruing may help to mitigate to some degree the odium heretofore cast upon that much-maligned little worker, the micro-organism.

Articles like Grosvenor's in the *Century*, and other papers on medico-scientific subjects, that have lately been printed in other high-class magazines, are a good thing for the lay reader, and the avidity with which they have been read and studied shows conclusively the interest of the people at large in all that pertains to their physical and material well-being.—*Lancet-Clinic.*

